# Expert agreement confirms that negative changes in hand and foot radiographs are a surrogate for repair in patients with rheumatoid arthritis

**DOI:** 10.1186/ar2220

**Published:** 2007-07-02

**Authors:** Désirée van der Heijde, Robert Landewé, Annelies Boonen, Steve Einstein, Gertraud Herborn, Rolf Rau, Siegfried Wassenberg, Barbara N Weissman, Carl S Winalski, John T Sharp

**Affiliations:** 1 Department of Rheumatology, Leiden University Medical Center, PO Box 9600, Leiden 2300 RC, The Netherlands; 2 Department of Rheumatology, University Hospital Maastricht, and CAPHRI Research Institute, PO Box 5800, Maastricht 6202 AZ, The Netherlands; 3 BioImaging Technologies, 826 Newtown-Yardley Road, Newtown, PA 18940, USA; 4 Department of Rheumatology, Evangelisches Fachkrankenhaus Ratingen, Rosenstrasse 2, Ratingen D-40882, Germany; 5 Department of Radiology, Brigham & Women's Hospital, 75 Francis Street, Boston, MA 02115, USA; 6 Department of Radiology, The Cleveland Clinic Foundation, 9500 Euclid Avenue, Cleveland, OH 44195, USA; 7 Department of Rheumatology, University of Washington, 8387 NE Sunamee Place, Bainbridge Island, WA 98110, USA

## Abstract

The objective of the present study was to test the hypothesis that experts recognize repair of erosions and, if so, to determine which, if any, morphologic features permitted them to recognize the repair. We also tested whether scoring by a standard method detected repair. Seven experienced readers of radiographs in rheumatoid arthritis were presented with 64 sets of single joints-of-interest at two time points, randomized and blinded for the correct sequence. The readers assessed which joint was better, and recorded whether any of six specific features were seen. Two independent readers, experienced in scoring by the van der Heijde-modified Sharp method who were not on the expert panel, then scored the complete films that included the joint-of-interest. The panel agreed very well on which of two joints was better, and, even though they did not know the true sequence, the panel accurately assigned a sequence slightly better than chance alone (58%) but worse than their agreement on which image was 'better or worse' (78%). The readers therefore indirectly assigned repair by choosing the second film as the best. Putative repair features were seen in cases of both repair and progression, and were not discriminatory. Similar results were obtained when the experts were presented with the entire hand or foot containing the joint-of-interest. In the third repair exercise, two independent readers who scored whole hands and feet using a standard method found a mean negative score in 22/60 joints-of-interest. All 22 joints were also scored as repair by the panel. Repair was detected reliably by a majority of the panel on viewing paired images based on a better/worse decision and assigning sequence in a set of images that were blinded for sequence by an independent project manager. In this test set of images, repair was manifested by a reduction in the size of erosion in many cases. Size was one feature that aided the experts to detect repair but cannot be the only one; the experts had to find other features to determine whether a smaller erosion was the first in a sequence of radiographs in a patient with progressive damage or was the second film in a patient exhibiting repair. The change in size of erosion was also picked up by independent readers applying the van der Heijde-modified Sharp scoring method and was reflected in their scores.

## Introduction

Damage of bone and cartilage caused by rheumatoid arthritis is visualized on radiographs as erosions and joint space narrowing. The focus of assessment until recently was on progression of damage. The first evidence that drug therapy might influence the course of rheumatoid arthritis appeared more than 30 years ago after the development of a method for scoring these abnormalities [1]. A decade and a half ago additional data became available to validate the term 'disease modifying antirheumatic drugs' when sulphasalazine was shown to slow radiographic progression [2]. Around the turn of the century it became obvious that radiographic progression could be stopped completely by current therapy in a large proportion of patients followed for 1–5 years, and it was appreciated that a number of patients had lower erosion scores in follow-up films [3,4]. During the same time period scattered reports were appearing of repair of erosions, many with equivocal supporting evidence [5-8]. Although a few studies presented images that were convincing, no studies have been performed to eliminate reader bias or to examine whether there are specific structural features that permit recognition of repair when the reader is unaware of the true sequence of films. The observation of negative scores, together with credible reports that healing might be real, posed the question of whether negative scores were detecting real structural improvement or artefact and, if real, whether the observations were clinically relevant or were trivial

There are additional reasons why these reports of healing and the negative scores drew attention. Of particular importance, therapy with TNFα blockers – which are more potent than previously employed therapies – resulted in lower levels of disease activity than were previously seen. As inflammation is a major driver of damage, an absence of inflammation *a priori *is a prerequisite for halting progression, and for making possible reversal of damage. In addition, data have been presented suggesting that TNFα blockers inhibit radiographic progression even in the presence of some persistent inflammation [9]. Presenting radiographic data by probability plots also revealed much more clearly the number of patients with negative scores by presenting the individual data of all patients, and this provided new insight into the potential magnitude of repair [10].

There is currently one trial in which a relatively large number of patients with negative erosion scores supports repair occurring on a group level [4]. Although the appraisal of repair on a group level is a relatively simple statistical matter, translating repair from the group level to an individual patient is not straightforward. The null hypothesis that there is no change from baseline within the group can be rejected if the mean change is below zero and the entire 95% confidence limit is below zero, which occurred in the TEMPO trial [11]. In contrast, a negative change score in an individual patient can be composed of 'true repair', of measurement error or of an image artefact such as rotation hiding a tangential erosion. The interrelationship of these three components is unknown and different in each patient. This argument is also pertinent in evaluation progression scores.

In a preliminary study within the OMERACT working group on repair we investigated whether a group of experts would agree on the presence of repair in a set of individual joints [12,13]. The conclusions were that repair indeed exists according to the majority judgements of the panel, but when the time sequence was blinded it was not possible for expert readers to distinguish cases with progression from cases with repair based on specific features considered relevant to repair, such as sclerosis, recortication, and filling-in of erosions. In that study the experts demonstrated good agreement on which image showed the least damage, but not on whether the best image was the first or second in time; in other words, whether the case was one of progression or repair. A few explanations for this observation were possible. First, there were quite different levels of experience among the readers, raising the possibility that the readers were not sufficiently experienced to recognize the features of repair. It was also possible that the images used in that study did not have a sufficient number of features of repair, or that the repair features were not clearly defined for technical reasons. Third, only single joints were presented to the readers, which might have hampered the correct ordering of the images into cases of progression or repair as change in other joints was not available to help in the decision. Most importantly, we were still not informed about the relation between cases depicted as repair by experts and negative scores obtained with traditional scoring methods such as the modified Sharp method [2,14].

We therefore embarked on three new exercises. First, we repeated the exercise with the single joints using a completely new set of images employing a larger number of images of better quality. In addition, we held a training session using cases not employed in the subsequent exercises that demonstrated repair as collected by one expert in the group (RR). In the second of the new exercises, we presented to the experts the entire hand or foot that included the joint-of-interest from the single joint exercise. This allowed us to test whether the presentation of the entire hand or foot improved the judgement of the correct time order of the films, thus indirectly labelling a case as progression or repair. Finally, we presented the entire hand and feet images to two independent readers who were unaware of the purpose of the reading when they scored the images by the van der Heijde-modified Sharp method. This third exercise tested the ability of readers to identify cases that included joints exhibiting repair and to link the scores of individual joints to those labelled as progression or repair by the experts [2].

## Methods

Eight experts, all experienced readers of rheumatoid arthritis radiographs, met for 3 days at Bio-Imaging Technologies (Newtown, NJ, USA).

The meeting started with a thorough training session discussing many examples of joints and features showing repair. These features were filling-in of erosions, recortication, sclerosis, remodelling, reconstitution of a normal joint, and trabeculation. The definitions were refined as compared with the previous exercise, and trabeculation was added as a feature that can help in distinguishing progression from repair. Filling-in, although clearly a reduction in the size of erosions, was thought by some to have additional information. Because recortication implies that the reader has concluded that the case is one of healing, in conducting the exercise readers recorded cortication of erosions and noted whether this was better or worse in the paired individual joints without regard to whether the reader had an opinion as to whether the pair showed progression or repair. It was also recognized that reconstitution of normal structure required a prior judgement as to whether repair was present.

Two exercises were performed thereafter on two consecutive days. The third exercise was performed separately by two readers not involved in the first two exercises.

Images for all three exercises were selected by one of the experts (JTS), who did not participate in the assessments, from a large set of radiographs available in digitized form from several data sources. Sixty-four image sets were selected, knowing the time sequence; approximately equal numbers of cases with repair, progression and equivocal or no change were included

### Exercise I

Cropped images of hands or feet containing the joint-of-interest with one or two adjacent joints to allow the reader to evaluate a change in radiographic positioning were selected. Images from two time points were paired, randomized, and blinded for sequence, and were presented to each reader independently on a reading station consisting of high-resolution monitors linked to a computerized data recording system. Readers were asked to choose the film that was worse or to declare no change, to choose which erosion was larger or to choose no change, and to choose which film was first in sequence or state unable to judge. In addition, readers recorded the presence of specific features of repair in one or both images. In the analysis, agreement was defined as concurrence of at least five of seven readers and was assessed for the better/worse/no change, the erosion size, and sequence decisions. Subsequently, the judgement of the individual panel member as to which joint was worse combined with that member's assignment of sequence provided an inferred choice of progression or repair, and was compared with the true sequence of the films in order to determine the accuracy of the assignment of progression or repair. The reader's assignment of progression was therefore a combination of the reader's choice of the better image with the choice of first in time or the combination of the worse image and the second in time; assignment of repair occurred with the choice of the better image and the second in time or with the choice of the worse image and the first in time (see Table 1).

**Table 1:** Study decision tree

Reader judged image A	Combined with true sequence of image A^b^	Conclusion in analysis^b^
Better^a^	First time point	Progression
Better	Second time point	Repair
Better and first time point	First time point	Reader recognized progression
Better and first time point	Second time point	Reader failed to recognize repair
Better and second time point	Second time point	Reader recognized repair
Better and second time point	First time point	Reader failed to recognize progression

The true sequence was unknown to the reader.

^a^A similar decision tree constructed for a reader judging image A as worse exchanges repair and progression in the conclusion column.

^b^In Exercises I and II, when the reader made a judgement as to whether a pair of images represented progression or repair that decision was called direct assignment. In the exercises when the analyst interpreted a reader's combined responses on better/worse and the sequence of images, this is called an inferred or an indirect assignment.

All observations of individual readers were pooled and the specific features were related to the progression and repair assessment. In total, seven readers provided 448 judgements of sets of two films. Of these 448 observations, 397 were considered to show change (repair or progression). These 397 observations were the basis for further testing the performance of single features of repair for detecting repair, defined as an appropriate decision about which film was the better in relation to the true sequence (least damage on the true second film). Odds ratios for the specific features for detecting repair in comparison with progression were calculated, as well as the sensitivity, the positive predictive value, the specificity, and the positive and negative likelihood ratios of these features.

One-third of the cases were selected as stable in the opinion of the selector (JTS) who is known as conservative in assessing change. In order to check the robustness of the main results, the analyses were repeated excluding such cases. The results confirmed and strengthened the conclusions reached by the primary analyses (data not shown).

### Exercise II

During the third day the same readers conducted the second exercise, in which they were presented with the entire hand or foot image that included the joints presented in the single joint experiment. The images were again randomized and recoded so they were not presented in the same sequence as for the first exercise, and the readers were not informed as to which joints had been presented in the earlier exercise, although some changes may have been sufficiently distinctive to enable the readers to remember from the exercise performed the day before.

Initially the experts were asked to judge the entire image as to which image was better, and whether the difference was due to progression or repair; in other words to make a direct judgement as opposed to the inferred judgement in Exercise I. The joint-of-interest in the first exercise was then indicated to the readers, and they repeated their review and chose which joint was better, and which film was first in time, to make possible a second inferred assignment of repair or progression. Panel agreement (at least five out of seven) was determined for the progression/repair judgement based on the whole hand/foot direct assignment and for the better/worse/no change judgement of the joint of interest. Judgement of the whole hand assignment of progression or repair was compared with the single joint inferred assignment of progression or repair.

The judgements of the single joints of Exercise II were then compared with the judgements of the single joints of Exercise I. Inter-reader agreement for Exercise II was assessed for each reader pair for both the single joint inferred assignment of repair and progression and the whole hand/foot direct assignment. All analyses on agreement were carried out with unweighted kappa statistics.

### Exercise III

Complete sets of hands and feet were available for 60 cases included in the first and second exercise, and these sets were presented with a blinded time sequence to two readers experienced in scoring rheumatoid arthritis films for trials but not involved in any of the exercises or discussions on repair. Readers were not aware of the fact that these images were part of a study to assess repair.

Films were scored by the van der Heijde-modified Sharp method [2]. Total scores were calculated (sum of erosions and joint space narrowing of hands and feet) for both readers. Average scores of the two readers were used for further analysis to mimic the situation in scoring clinical trials. Readers' scores for the joints-of-interest and for the total score were compared with the panel judgement.

## Results

### Exercise I

The readers agreed on which film was better, worse or no change in 77% of the cases. Agreement was similar for erosion size (78%) and better than for the correct sequence (58%). The readers therefore agreed on which individual joints showed the least damage, and their single joint inferred assignment of progression or repair was slightly greater than expected by chance alone. Taking all of the assignments of all readers separately, a reader assigned 'no change' to a pair of films 51 times – indicating that the readers were more willing than the project manager who selected the cases to assign a better or worse status than no change. An inferred assignment of repair was made 254 times, and progression was assigned 143 times, which gives us the prior probability of repair (64%).

Table 2 presents the number of observations (all observations were pooled) in which single features of repair were scored as being present. Only the 397 cases in which the readers scored change were taken into account in this analysis. Features are ordered by decreasing prevalence and sensitivity to detect repair. The most frequently observed feature was filling-in of erosions (337/397), followed by cortication (276/397), sclerosis (217/397), remodelling (129/397), trabeculation (119/397), and reconstitution of a normal joint (78/397). The odds ratios for filling-in of erosions, cortication, sclerosis, and remodelling suggest a more frequent recognition of these features in repair cases. Despite odds ratios > 1, the discriminatory capacity of a single repair feature in distinguishing between repair and progression was very low, as deduced from the positive predictive values in comparison with the prior probability of repair in this set (64%), and from the likelihood ratios. For example, the highest odds ratio (2.7) is for filling-in of erosions, which is equivalent to reduction in the size of erosions. In those cases in which 'filling-in of erosion' was considered present, however, only 67% of the cases were given a single joint inferred assignment of repair, as compared with the prior probability of 64%. This is reflected by a very low positive likelihood ratio of 1.1 and a rather high negative likelihood ratio of 0.50. In contrast to the first five listed features (filling-in, cortication, sclerosis, remodelling, and trabeculation), reconstitution of the normal structure was recorded more frequently in progression cases. Specificity was less than 0.8 in all cases. Sensitivity was less than 0.6 for four of the six features: filling-in of erosions performed badly because of lack of specificity; sclerosis, remodelling, trabeculation, and reconstitution failed because of lack of sensitivity.

**Table 2:** Results of specific repair features in Exercise I^a^

Single repair feature	First film is better^b ^(progression) (*n *= 143, 36%)	Second film is better (repair) (*n *= 254, 64%)	Odds ratio to detect repair	True positive rate (sensitivity)	False positive rate (1 – specificity)	Positive likelihood ratio	Negative likelihood ratio
Filling-in of erosions	112 (33%)*	225 (67%)**	2.2	0.89	0.78	1.1	0.50
Cortication	84 (30%)	192 (70%)	2.2	0.76	0.59	1.3	0.58
Sclerosis	68 (31%)	149 (69%)	1.6	0.59	0.47	1.1	0.78
Remodelling	32 (24%)	97 (76%)	2.1	0.38	0.22	1.7	0.79
Trabeculation	41 (37%)	78 (63%)	1.1	0.20	0.21	1.0	1.0
Reconstitution of a normal joint	41 (53%)	37 (47%)	0.43	0.15	0.29	0.5	1.2
Any of the above features of repair	130 (36%)	234 (64%)	1.2	0.92	0.91	1.0	0.89

^a^Test performance in Exercise I of putative features of repair in relation to progression and repair as indicated by inferred assignment (first film is better versus second film is better). Of the total number of 397 observations, 143 (36%) were judged as showing progression and 254 (64%) as showing repair without taking into account specific features of repair. Adding information on features of repair only marginally influences the discrimination between progression and repair.

^b^Designation of first or second film based on the true sequence. Numbers indicate the numbers of observations in which a given single repair feature was recorded as present. Percentages indicate the positive predictive value of a specific repair feature for a progression* or repair** classification. For example, 'filling-in' was observed 325 times: 103 times (32%) in cases with progression and 222 times (68%) in cases of repair. The positive likelihood ratio is the quotient of the true positive rate divided by the false positive rate (for example, 'filling-in' in cases of repair) and the false positive rate (for example, 'filling-in' in cases of progression). The negative likelihood ratio is the quotient of the false negative rate (no 'filling-in' in cases of repair) divided by the true negative rate (no 'filling-in' in cases of progression). In order to be of diagnostic value, the positive likelihood ratio should be high (for example, > 4) and the negative likelihood ratio should be low (for example, < 0.3).

The contribution of specific features to detect repair was also checked to determine whether detecting the feature was dependent on the true sequence in which the images were presented to the reader. Overall, such an effect could not be demonstrated (data not shown).

### Exercise II

For this exercise we calculated the kappa statistic (κ) for each reader-pair. The mean (standard deviation) κ value, computed across all possible reader-pairs, was 0.52 (0.10) for the inferred progression/repair/no change assignment, based on a better/worse decision. The mean (standard deviation) κ value for a whole hand/foot direct assignment of progression/repair/no change decision, however, was significantly lower (0.34 (0.09)); the paired *t *value for the difference between indirect and direct assignments was -6.3 (*P *< 0.001)). This finding is again compatible with readers agreeing on which film is better (size of erosions), but agreeing less well on whether such a difference is due to repair or progression. Implicitly, this finding also underscores that there are no typical features regularly recognized by all readers pointing to repair, confirming what was shown in Exercise I.

Offering the entire hand or foot resulted in an agreement (≥ 5/7 readers agreed) on an inferred progression/repair assignment in 53/64 (88%) patients. Agreement on a direct whole hand/foot repair/progression/no change assignment occurred in 42/64 (66%) patients. In the single joint experiment, these figures for inferred assignment were 77% and 58%, respectively.

In the whole hand direct assignment, the panel judged only nine cases as 'repair' (Table 3). All these cases were assigned repair by inferred assignment in the same exercise. If the inferred assignment (28 cases of repair) was considered the gold standard, however, the panel missed 19 of these cases in their whole hand/foot direct assignment: eight of the missed cases were judged as progression and 11 cases did not reach a majority agreement.

**Table 3:** Assignment of progression or repair based on direct assignment versus inferred assignment in Exercise II^a^

Whole hand direct assignment of progression/repair	Single joint inferred assignment of progression/repair				
	
	Total	First film is better (progression)	Second film is better (repair)	Both films are similar (no change)	No majority agreement obtained
Progression	28	18	8	0	2
Repair	9	0	9	0	0
No change	5	0	0	4	1
No majority agreement obtained	22	3	11	0	8

Total	64	21	28	4	11

^a^Based on agreement by five of seven readers.

For each reader we compared the direct assignment of repair with the inferred assignment, using the inferred assignment as the gold standard because this only involves one decision about better/worse. All experts' scores were remarkably similar with respect to assigning progression or repair, except for one reader. A typical example of results of a single reader is presented in Table 4. The percentages of correctly classified cases (agreement between direct whole hand/foot image and inferred assignment) ranged for all readers from 70% to 75%. The percentages of cases falsely classified as having repair ranged from 1.5% to 5%, and those of cases falsely classified as no repair ranged from 22% to 25% for six of the seven readers. The remaining reader scored repair much more frequently than the other readers, but classified 11% falsely as repair and 14% falsely as progression. The positive predictive value of a direct repair assignment ranged from 80% to 96% and the negative predictive value from 56% to 69%, with the reader scoring more repair as having the highest negative predictive value and the lowest positive predictive value. These data are compatible with a conclusion that experts underperform with respect to repair if they do not know the true time order. They indirectly see repair because they see change, but they do not directly recognize it as such.

**Table 4:** Typical example of the direct versus the inferred assignment of one of the readers

Reader's direct judgement (direct assignment)	Reader's better/worse interpretation in combination with true time order of X-rays (inferred assignment):		Totals
		
	Compatible with progression or no change	Compatible with repair	
Progression/no change	21	15	36
Repair	1	27	28

Totals	22	42	64

The inferred assignment is considered the gold standard. Direct assignment underestimates the true prevalence of repair. Percentage correctly classified, 75%; false direct assignment of repair, 1.5%; false direct assignment of progression, 23%; positive predictive value, 96%; negative predictive value, 58%.

After the judgement of the whole hand or foot, without knowledge of the joint-of-interest, we unblinded the joint-of-interest and again performed an indirect assignment of repair; we then compared these results with the results of the indirect assignment of the single joint experiment (Exercise I), both at the level of the individual experts and at the level of the panel. Absolute intra-reader agreement varied from 74% to 87% (κ = 0.52–0.74, indicating moderate to good agreement). Panel agreement (majority decision of at least five out of seven readers) with regard to the first and the second assignment was 85% (κ = 0.69).

### Exercise III

**Figure 1:**
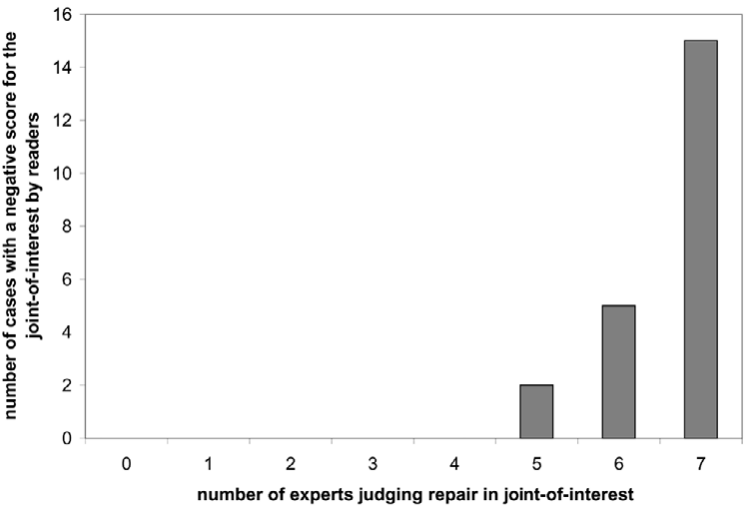
Agreement between negative van der Heijde-modified Sharp scores and the expert panel judgement on repair. Comparison of the number of cases with a negative mean score of the van der Heijde-modified Sharp score by two readers for the joint-of-interest (total *n *= 22) with the numbers of experts (total *n *= 7) assessing the joint as showing repair.

The complete sets of hand and foot films of the 60 patients that included 64 joints-of-interest (four patients had more than one joint of interest) presented to the experts in Exercises I and II were scored by the van der Heijde-modified Sharp method; two experienced readers gave a mean negative score to 22 of these joints-of-interest. Figure 1 shows that all cases with a negative joint-of-interest score by these readers were confirmed by a majority (≥ 5/7) of the experts as repair in Exercise I. In 15 of these 22 joints-of-interest with a negative change score there was complete panel agreement.

Twenty-three patients were given a mean negative change score on the overall score for both the hands and feet. Seventeen of these patients were judged to show repair by the experts viewing only the joint-of-interest in Exercise I; in the remaining six cases, the two independent readers did not score repair in the joint-of-interest, which was in agreement with the judgement of the expert panel in all cases. In seven cases there was agreement between both independent readers and the experts that the joint-of-interest showed repair, but the score for both hands and feet demonstrated progression.

In every case, repair judgement was based on improvement in erosions, and not on improvement in joint space narrowing. In contrast, progression in joints-of-interest appeared to be the consequence both of progression in erosions and of joint space narrowing.

## Discussion

The logic incorporated in the study design in Exercises I and II, illustrated in Table 1, was critical to the analysis and to the conclusions reached. If repair is a reality and alters the bone structure in a distinctive and recognizable way, when experts view two images of the same joint or of the entire hands or feet that are randomly ordered for sequence they should be able to tell which image includes the repaired bone, provided that the technical factors in capturing the image are identical. It is entirely possible in the early stages of developing an erosion that, if the inflammatory process is completely halted, the erosion might heal and leave no structural changes indicative of the healing process. These cases would not be recognized by an expert under the circumstances of Exercises I or II because of the randomization and blinding of the sequence, but would be detected by the standard scoring procedures. In cases that were characterized by morphologic features of repair, combining the individual's judgement regarding better/worse with the true sequence allowed the analyst to infer whether repair or progression of the erosion has occurred in the interval between the two images. It is critical to mention here that a conclusion of repair or progression can be reached irrespective of the reader's opinion. In other words, readers' bias has been eliminated.

Results from both Exercises I and II show that readers agree quite well on which of two images of single joints is the better, and on which shows the smaller erosion. Agreement was improved when the cases selected as stable or questionable and those selected as probable but not definite repair or progression were omitted. This indicated that readers were able to accurately recognize a single feature or a combination of features that was interpreted as repair in many cases. Assignment of the correct sequence was significantly greater than by chance alone when these cases were omitted from the analysis. The analysis that included all the cases more closely reflects reality in clinical studies since there will always be cases that are equivocal. The analysis that excluded these cases indicates that where there is a clear-cut difference between images at two time points at intervals of 6 months to 5 years, the panel's agreement as to which is better and which is the first image in the interval is better than chance alone – indicating that there are single features or combination of features that are recognizable and indicate repair.

Assignment of the correct sequence is only marginally improved when the experts view the entire hand or foot film. The association of most of the signs of repair in sets of single joint images with repair is hardly better than expected by chance alone. When blinded to sequence, experts frequently and inadvertently adjudicated signs of repair to sets of images that actually show progression. Based on these results, single features of repair should not be included in radiographic scoring methods. The relatively low kappa values for intra-reader and inter-reader agreement indicate that basing the assignment of repair on the judgement of only one expert is not reliable. The panel also performed considerably better, however, in the test-retest situation if a judgement of repair was based on a majority decision of at least five out of seven readers. Although the decision to use concurrence by five or more of the seven readers as a definition of 'agreement' was an *a priori *one, the analysis indicates that conclusions based on 5/7 agreement in this analysis are conservative.

In Exercise III two independent readers that regularly score films of hands and feet according to the van der Heijde-modified Sharp method, without knowledge of time sequence, provided negative change scores in individual joints that were judged as repair by the expert panel in Exercises I and II. In all cases, this was due to improvement in erosions, not to improvement in joint space narrowing. The picture, however, is more complicated. Among patients with a positive change in total Sharp scores, we found cases of negative erosion scores in joints-of-interest that were confirmed as repair by the expert panel. There are two possible interpretations for this observation: overall progression of damage does not preclude repair in single joints, or technical factors create apparent improvement (that is, improvement is not real). For example, a change in radiographic positioning of the joint, a different dynamic range between the two films, a change in soft tissue during the interval and possibly other factors may produce spurious changes.

What clearly emerges from these findings is that experts quite regularly agree on which image is better. Based on this study, if we assume that the image is an accurate representation of the true damage, repair is a reality and this observation confirms and extends our previous findings. In another study by Rau and colleagues, 74 joints out of 1,292 joints showed repair phenomena [7]. The authors also found that, in the group of patients with repair phenomena in single joints, an increase and decrease in the score occurred in different joints in the same patient at the same time.

The net change score not only reflects numbers of joints, but also the magnitude of change per joint. If, in an individual patient, the joints with negative change scores (repair) outweigh the joints with positive change scores (progression), the total Sharp change score, which is the sum of all individual joint scores, will become negative. It is likely that the magnitude of change per joint is higher in cases of progression as compared with repair, since repair is usually subtle and may be limited in extent, whereas individual joint progression can be extensive and can easily involve two or more scoring units. Moreover, repair in the individual patient is constrained by the number of joints with damage, and probably also by the level of damage in those joints. In fact, data from animal studies clearly indicate that once the matrix is resorbed the rate and extent of depositing new matrix is limited, which in turn limits the extent of reconstruction of bone. In contrast, progression can occur in both damaged and undamaged joints. Scoring methods therefore cannot capture every individual demonstrating repair in one or more joints; it is also true that scoring cannot capture every case of progression. It also is considered very probable that healing may occur in the minimally damaged joint without leaving any trace of prior damage or distinctive features in the reconstituted bone. Under these circumstances, even though only the presence/absence of the erosion indicates that repair has occurred, the healing process would be reflected in the score.

The net change score also reflects the measurement error and anticipation bias. The former includes the true measurement error, which includes reading error and error invoked by changes in radiographic positioning and exposure. Anticipation bias may arise when readers are influenced by the status of other joints in the same image and inaccurately score one or more individual joints; for example, a questionable new erosion becomes much more definite to the reader if several other joints show clear-cut progression.

In the individual patient it is impossible to judge how and to what extent measurement error and anticipation bias contribute to the score, negative or positive. The more negative a score that includes scores of 44 or more joints for a patient, the greater the likelihood that repair has occurred at least in some joints. If in a group of patients the effect of negative scores outweighs the effect of positive scores, the group change will become negative. This balance incorporates the number of patients as well as magnitude of change. Since it can be expected that, in terms of magnitude, positive change will outweigh negative change, the number of patients with a negative score has to be higher than the number of patients with a positive score before the group change will become negative. We therefore conclude that a negative change for an entire group of patients, for example a treatment arm in a therapeutic trial, may be a very conservative estimate of the existence of repair in single joints. A firm conclusion therefore seems justified: the more negative a group change, the higher the total number of single joints with a negative joint score, and the probability that true repair has contributed to these negative scores is greater. These arguments clearly demonstrate how difficult it is to translate negative group change to repair in a single joint.

In analysing the data of the present study we have seriously considered whether traditional scoring methods are sufficient to pick up joints with repair, or whether specific features of repair should be incorporated in the scoring method to improve detection of repair. Provided recognition of features of repair were highly reproducible, incorporating them in a scoring system would improve recognition of repair, particularly in those cases in which both repair and progression is observed. Based on the poor performance of the specific features as indicated by the very low likelihood ratios, however, it would not be advantageous to include them in the scoring methods at present. But this should not be considered a closed issue; a more standardized radiographic technique to reduce imaging artefact and more training of readers might improve sensitivity and reliability of detecting repair.

The present study has shown that a reduction in the score reflects repair, and, although we are unable to assess how many cases of repair could not be captured by scoring, as stated above, the current state of the art does not suggest that recording presence of features of repair would significantly improve their capture.

## Conclusion

The results of the three exercises combined lead to the following conclusions. Repair does exist; a majority of a panel of experts judged the follow-up image to be better when presented with single joints from each time point, the pair having been selected for illustrating repair, even though the images were blinded as to sequence and were randomly ordered and mixed with cases of progression and equivocal or no change when presented to the readers. Furthermore when the panel was shown the entire hand or foot film in a separate session that included the joints selected as demonstrating repair, the panel again selected the second in the true order as improved. Recording the presence of specific features of repair was not consistent or sensitive enough to recommend incorporation in scoring methods. In the present study the most frequently recorded feature indicating repair was a reduction in the size of existing erosions. This 'negative progression' was also picked up by readers applying a standard scoring method who were not aware that they were seeing repair because the time order was concealed.

## Abbreviations

TNF = tumour necrosis factor.

## Competing interests

The authors declare that they have no competing interests.

## Authors' contributions

DvdH, RL, SE, and JTS participated in the design of the study. DvdH, AB, GH, RR, SW, BNW, and CSW scored joints-of-interest. JTS selected the images for the exercises. RL conducted the statistical analyses. DvdH, RL, and JTS interpreted the results. DvdH, JTS, and RL drafted the manuscript. All authors read and approved the final manuscript.
